# News from Societies

**Published:** 1958-10

**Authors:** 


### *News from Societies*

### PROGRAMMES FOR 1958–59 SESSION

### BRISTOL MEDICO-CHIRURGICAL SOCIETY

*Secretary:* Dr. B. E. McConnell, 4 Napier Road, Bristol 6

8th Oct.: **Annual General Meeting. Presidential Address.** H. L. Shepherd, Esq., CH.M., F.R.C.O.G.
12th. Nov.: **Clinical Meeting.** Bristol General Hospital.
10th Dec.: Professor R. W. Scarff, F.R.S.ED., **"Breast Cancer".**
14th Jan.: Leslie C. Hill, Esq., M.D., F.R.C.P., **"Pain".**
11th Feb.: Sir Walter Mercer, F.R.C.S.ED., **"Shoulder Pain".**
11th Mar.: H. Gordon Mather, Esq., M.R.C.P., **"Cardiac Iscaemia".**
8th Apr.: Kenneth G. Malcolmson, Esq., F.R.C.S., **"Rhinitis".**
13th May: T. J. Butler, F.R.C.S., and W. M. Capper, Esq., F.R.C.S., **"Aftermath of Gastrectomy".**

### THE DEVON AND EXETER MEDICO-CHIRURGICAL SOCIETY

*Secretary:* Dr. Charles Seward, 20 West Southernhay, Exeter

16th Oct., at 3.30 p.m.: Lambert Rogers, Esq., C.B.E., V.R.D., M.D., M.SC., F.R.C.S., F.R.A.C.S., F.A.C.S., **"Some Thoughts on Surgical Progress".**
6th Nov., at 3 p.m.: **Clinical Meeting.**
28th Nov., at 8.15 p.m.: Robert Nevin, Esq., T.D., M.A., F.R.C.S., **"The Surgery of Ulcerative Colitis".**
11th Dec., at 8.15 p.m.: **Clinico-Pathological Demonstration.**
8th Jan., at 8.15 p.m.: President's Party for Members and their wives in Board Room, Royal Devon and Exeter Hospital. Film: **"A Two-year old Goes to Hospital"**, in Medical Library.
29th Jan., at 8.15 p.m.: **Clinical Meeting** at P.E.O.H.
9th Feb., at 8.15 p.m.: Lewis Couper, Esq., M.B., CH.B., D.P.M., **"The Psychosomatic Approach".**
12th Mar., at 3 p.m.: **Clinical Meeting.**
9th Apr., at 8.15 p.m.: **Annual General Meeting and Clinico-Pathological Demonstation.**

### WEST OF ENGLAND CHILD HEALTH GROUP

*Secretary:* Dr. P. G. Roads, Central Health Clinic, Tower Hill, Bristol 2

Meetings at the Children's Hospital, Bristol.
21st Oct., at 8.30 p.m.: Miss Doris Odlum, M.A., M.R.C.S., D.P.M., **"Adolescence".**
6th Nov., at 8.30 p.m.: E. Rogan, Esq., L.R.C.P.I., D.P.H., **"Age of the Menarche".**
9th Dec., at 8.30 p.m.: Professor A. V. Neale, **"Paediatrics in the South Pacific".**
15th Jan., at 8.30 p.m.: Professor A. I. Darling, **"Preventive Dentistry".**
5th Feb., at 5.30 p.m.: David M. Stevens, Esq., M.B., F.R.C.S., D.L.O., **"Catarrhal Deafness in Childhood".**
12th Mar., at 8.30 p.m.: P. R. Evans, Esq., M.D., M.SC., F.R.C.P., **"Baby Farming".**
May (date to be announced later): Miss Cecile Hopkins, M.R.C.S., L.R.C.P., D.P.H., **"Problem Families".**

### B.M.A. (Barnstaple Division)

*Secretary:* Dr. S. G. Brook, Eddleston, Park Lane, Barnstaple

1st Oct., at 8.30 p.m.: **Clinico-Pathological Meeting** at the North Devon Infirmary, arranged by Dr. R. Tierney. (Preceded by supper at 7.30 p.m.)
1st Nov., at 8.30 p.m.: H. L. Shepherd, Esq., CH.M., F.R.C.O.G. and G. R. Airth, Esq., M.B., B.S., D.M.R.D., **"The Use of X-rays in Pregnancy"**, at the North Devon Infirmary. (Preceded by supper at 7.30 p.m.)
Dec. (on a date to be arranged): A Farewell Dinner to S. C. Shaw, Esq., F.R.C.S., on his retirement.
Other meetings yet to be arranged include a Clinical Meeting at Bideford Hospital, an address on "Modern Therapeutics" and a Cocktail Party to welcome new members.

### B.M.A. (Bristol Division)

*Secretaries:* Dr. A. S. Anderson, 1 Dean Lane, Bristol 3, and
Dr. Sarah Walker, 44 Kewstoke Road, Bristol 9

7th Oct., at 8 p.m.: Donald Hunter, Esq., C.B.E., M.D., F.R.C.P., **"New Problems in Public Health"** (Sir Charles Hastings Lecture 1958), Victoria Rooms. This lecture is open to the general public.

19th Oct., at 2.45 p.m.: St. Luke's Tide Service in Bristol Cathedral. Preacher: The Very Rev. D. E. W. Harrison, Dean of Bristol.

21st Nov.: **Annual Dance,** Grand Spa Hotel, Clifton.

21st Jan., at 8.30 p.m.: Allan F. Rogers, Esq., M.D., **"The Antarctic Expedition".** At the Royal Fort.

6th May, at 8.30 p.m.: **Annual General Meeting** at the Royal Fort.

27th May, at 8.30 p.m. (provisional date): **Clinical Meeting** at Southmead Hospital.

12th June, at 4 p.m. (provisional date): Reception for newly qualified doctors. In the Garden Hall, the Royal Fort.

### B.M.A. (Cornwall Division)

*Secretary:* Dr. E. Townsend, Fieldways, Tregenna Lane, Camborne

1st Nov., at 2.30 p.m.: **Clinical Meeting,** Royal Cornwall Infirmary, Truro.

6th Nov., at 8.30 p.m.: H. Osmond-Clarke, Esq., C.B.E., F.R.C.S., **"Pain in the Head and Neck".** (B.M.A. Clinical Lecture.) Royal Cornwall Infirmary, Truro.

27th Nov.: **Annual Dinner and Dance,** Hotel Bristol, Newquay.

Apr. (details to be announced later): Sir Stanford Cade, K.B.E., C.B., F.R.C.S. Lecture on Radiology.

### B.M.A. (Gloucestershire Branch)

*Secretary:* Dr. H. G. Scott-Kerr, 29 Leckhampton Road, Cheltenham

*Cheltenham* meetings at the General Hospital and *Gloucester* meetings at Hut 6, City General Hospital, Great Western Road.

9th Oct.: R. Harvey, Esq., M.B.E., F.R.C.S., **"Doctor in Prison".** Presidential Address, Cheltenham.

13th Nov.: **Clinical Meeting,** Gloucester.

11th Dec.: D. B. E. Belson, Esq., Director of John Harvey & Sons, Ltd., Bristol, **"Wines and the Palate".** Film: "Talk and Tasting", Cheltenham.

8th Jan.: R. Belsey, Esq., M.S., F.R.C.S. will read a paper. Gloucester.

12th Feb.: **Clinical Meeting,** Cheltenham.

12th Mar.: H. Mower, Esq., M.R.C.S., L.R.C.P., D.L.O., **"Deafness",** with film of demonstration of cases. Gloucester.

9th Apr.: Sir Gordon Gordon-Taylor, K.B.E., C.B., M.D., F.R.C.S., **"In Retrospect".** B.M.A. Lecture, Cheltenham.

7th May: **Annual General Meeting.** F. B. P. Evans, Esq., M.B., B.S., **"Fads, Prejudices and Errors in General Practice".** Tewkesbury.

11th June: Address by President's guest. (This is in place of Mr. Stallard's paper which will not now be given.)

### B.M.A. (South-Western Branch)

*Secretary:* Dr. F. E. Graham-Bonnalie, St. John's House, 37 The Strand, Topsham, near Exeter

30th Oct., at 8.15 p.m.: Address by A. Lawrence Abel, Esq., M.S., F.R.C.S., Royal Devon and Exeter Hospital.

1st Nov., at 2.30 p.m.: **Clinical Meeting.**

### BRISTOL SOUTH MEDICAL GROUP

*Secretary:* Dr. H. I. Howard, Claremead, 102 Church Road, Bishopsworth, Bristol 3

Meetings take place on the first Sunday in each month during the winter at the Bristol General Hospital at 8.p.m.

### COSSHAM MEDICAL SOCIETY

*Secretary:* Dr. R. C. Wyldesley, 267 Soundwell Road, Kingswood, Bristol

Meetings at Cossham Hospital at 8.45 p.m.

14th Oct.: R. M. Courtney, Esq., M.B.CH.B., D.P.H. **Presidential Address.**

4th Nov.: R. T. Routledge, Esq., M.B., F.R.C.S., **"A Tale of Two Cities".**

2nd Dec.: R. Wohlfeld, Esq., M.D., **"Osteopathy—Pro and Contra".**

6th Jan.: Professor C. Bruce Perry, **"Iatrogenic Diseases".**

3rd Feb.: Donal Early, Esq., L.R.C.P.I., D.P.H., **"Anxiety States and their Treatment".**

3rd Mar.: J. A. Cosh, Esq., M.D., M.R.C.P., **"The Management of Hypertension".**

17th Mar.: **Annual Dance,** Ashton Court Country Club.

7th Apr.: **Annual General Meeting.**

### BATH CLINICAL SOCIETY

*Secretary:* Dr. J. R. Bolton, West Lodge, 61 Englishcombe Lane, Bath

All meetings will be held at 8 p.m. at the Royal United Hospital, Bath, except the November meeting which will be held at St. Martin's Hospital, Bath.

10th Oct.: H. Lovell Hoffman, Esq., M.D., F.R.C.P. and J. Montgomerie, Esq., M.B.CH.B., F.F.A.R.C.S., **"Management of Respiratory Complications of Neurological Diseases".**

14th Nov.: **Clinical Meeting.**

12th Dec.: Mrs. C. D. Cross, M.D., M.R.C.P., **"Treatment and Prognosis in Haemolytic Disease of the Newborn".**

9th Jan.: J. A. Cosh, Esq., M.D., M.R.C.P., C. P. Sames, Esq., M.S., F.R.C.S., A. C. P. Thomson, Esq., M.B.CH.B., D.M.R., G. H. Williamson, Esq., M.B.CH.B., **Symposium on Disorders of the Colon.**

13th Feb.: F. Kohn, Esq., M.D., **"Ignaz Semmelweis".**

13th Mar.: Norman Capon, Esq., M.D., F.R.C.P. Guest Speaker.

10th Apr.: **Clinical Meeting.**

25th Apr.: **Annual Dinner** at Fortt's Restaurant.

### CORNWALL CLINICAL SOCIETY

*Secretaries:* Dr. J. D. Hardy, Royal Cornwall Infirmary, Truro, and
Dr. B. A. Gwynne Jenkins, Central Chest Clinic, Tuckingmill, Cornwall

2nd Oct.: H. Osmond-Clarke, Esq., C.B.E., F.R.C.S., **"Pain in the Neck and Arm".** (B.M.A. Lecture.) Royal Cornwall Infirmary.

7th Nov.: **Clinical Meeting.** Camborne–Redruth Hospital.

27th Nov.: **B.M.A. Dinner and Dance.** Hotel Bristol, Newquay.

4th Dec.: **Clinical Meeting or Symposium.** Royal Cornwall Infirmary.

9th Jan.: Short Papers by General Practitioners, Camborne–Redruth Hospital.

6th Feb.: Sir William Porter MacArthur, K.C.B., D.S.O., O.B.E., **"Samuel Pepys and the London Plague".** Royal Cornwall Infirmary. (Ladies' Night.)

6th Mar.: **Clinical Meeting,** Chest Hospital, Tehidy.

Apr. (date to be announced later): **B.M.A. Lecture.** Royal Cornwall Infirmary.

7th May: **Cases and Demonstrations** by the Consultant Staff, Royal Cornwall Infirmary.

5th June: J. G. Hastings Ince, Esq., M.B., B.S., F.R.C.O.G. **Presidential Address. Annual General Meeing,** Camborne–Redruth Hospital.

All meetings at 8 p.m.

### ROYAL DEVON AND EXETER HOSPITAL POSTGRADUATE PROGRAMME

*Secretary:* Dr. Anthony Daly, 30 Barnfield Road, Exeter

The Exeter Postgraduate Meetings will take place on Tuesdays at 8.30 p.m. from 14th October to 9th December.

### SOCIETY OF MEDICAL OFFICERS OF HEALTH WEST OF ENGLAND BRANCH

*Secretary:* Dr. R. H. G. H. Denham, Bathavon Council Offices, 30 Westgate Buildings, Bath

The Society is divided into two sections, Northern and Southern. Each section holds four meetings a year, one of which being a joint meeting of both sections.

*Northern Section:*

4th Oct.: D. E. Cullington, Esq., M.A., M.B., B.CHIR., D.C.H., D.P.H. **Presidential Address.** At Taunton.

3rd Jan.: At Bristol (details not yet available).

4th Apr.: At the Pump Room, Bath (details not yet available).

May: Joint Meeting of Northern and Southern sections (details not yet available).

### SOUTH SOMERSET MEDICAL CLUB

*Secretary:* Dr. N. K. Dryden, 2 Penn Hill, Yeovil

16th Oct.: F. S. W. Brimblecombe, Esq., M.D., M.R.C.P., **"Common Problems in Paediatrics".**

20th Nov.: **Clinical Meeting.**

Jan. (date to be announced later): **Dinner and Dance.**

19th Feb.: **Paper** by J. F. Davidson, Esq., O.B.E., M.B.CH.B., D.P.H.

19th Mar.: **Symposium.**

18th Apr.: **Paper** by T. S. Wilson, Esq., M.D

### TORQUAY AND DISTRICT MEDICAL SOCIETY

*Secretaries:* Dr. D. K. MacTaggart, " Broadwater", Preston Down Road, Paignton, and Dr. I. F. R. Sutherland, 1 Shiphay Avenue, Torquay

Meetings are held at Torbay Hospital at 8.30 p.m. unless otherwise stated.
30th Oct.:
4 p.m. R. S. Corbett, Esq., M.CHIR., F.R.C.S., **"Ulcerative Colitis".** Torbay Hospital.
7.30 p.m. **Dinner and Dance** at the Palace Hotel, Torquay.
13th Nov.: Maurice Campbell, Esq., O.B.E., F.R.C.P., **"Treatment of Coronary Disease".**
19th Dec.: Allan F. Rogers, Esq., M.D., **"The Trans-Antarctic Expedition".**
29th Jan.: A. J. Rook, Esq., M.D., M.R.C.P., **"Eczema in Infancy".**
26th Feb.: Murray A. Falconer, Esq., M.CH., F.R.C.S., **"The Treatment of Malignant Disease by Hypophysectomy".**
12th Mar.: **Clinical Discussion—"Infertility"** opened by Mrs. Margaret Jackson, M.B., D.OBST., R.C.O.G.
30th Apr.: A. S. King, Esq., B.SC., PH.D., F.R.C.V.S., **"Diseases of the Invertebral Disc".** Joint meeting with the Western Counties Veterinary Association.
28th May: **Annual General Meeting.** Victoria Hotel, Torquay.

### WEST PENWITH MEDICAL SOCIETY

*Secretary:* Dr. Eileen E. Wood, West Cornwall Hospital, Penzance

16th Oct., at 8.15 p.m. **Annual Dinner.** Queen's Hotel, Penzance.
20th Nov.: T. S. Wilson, Esq., M.D., **"Restlessness and Mental Confusion in the Elderly".** Mrs. E. E. Wood, M.R.C.S., L.R.C.P., **"Anaemia in Old Age".**
18th Dec.: J. H. Willis, Esq., of Melbourne Botanical Gardens. **"The Australian Flora".** Illustrated by colour photographs. This is an open meeting to members' wives and friends.
15th Jan.: Mark Hewitt, Esq., M.B., B.S., M.R.C.P., **"Dermatological Problems and Cases".**
19th Feb.: R. Adlington, Esq., F.R.C.S., **"Vascular Emergencies".**
19th Mar.: **"Laboratory Aids to Diagnosis"** by the staff of West Cornwall Hospital Laboratory.
16th Apr.: **B.M.A. Lecture** by Sir Stanford Cade, K.B.E., C.B., F.R.C.S. Jointly with Cornwall Clinical Society and Cornwall Division of the B.M.A., Royal Cornwall Infirmary.
21st May: **Medico-Legal Session.**
18th June: **Clinical Meeting.**

